# Data Resource Profile: Primary Care Audit, Teaching and Research Open Network (Patron)

**DOI:** 10.1093/ije/dyae002

**Published:** 2024-02-01

**Authors:** Jo-Anne Manski-Nankervis, Rachel Canaway, Christine Chidgey, Jon Emery, Lena Sanci, Jane S Hocking, Sandra Davidson, Indi Swan, Dougie Boyle

**Affiliations:** Department of General Practice and Primary Care, Melbourne Medical School, University of Melbourne, Melbourne, VIC, Australia; Department of General Practice and Primary Care, Melbourne Medical School, University of Melbourne, Melbourne, VIC, Australia; Department of General Practice and Primary Care, Melbourne Medical School, University of Melbourne, Melbourne, VIC, Australia; Department of General Practice and Primary Care, Melbourne Medical School, University of Melbourne, Melbourne, VIC, Australia; Department of General Practice and Primary Care, Melbourne Medical School, University of Melbourne, Melbourne, VIC, Australia; Melbourne School of Population and Global Health, University of Melbourne, Carlton, VIC, Australia; Department of General Practice and Primary Care, Melbourne Medical School, University of Melbourne, Melbourne, VIC, Australia; Department of General Practice and Primary Care, Melbourne Medical School, University of Melbourne, Melbourne, VIC, Australia; Department of General Practice and Primary Care, Melbourne Medical School, University of Melbourne, Melbourne, VIC, Australia

**Keywords:** Dataset, primary care, epidemiology

Key Features

- The Primary Care Audit, Teaching and Research Open Network (Patron) is an Australian database of de-identified electronic medical records of primary care patients which can be used to inform research and policy questions from diverse fields including population health, health economics, health policy, general practice, pharmacoepidemiology and education.
- Patron was established by the University of Melbourne in partnership with general practices in a practice-based research network in 2016, and de-identified data sharing commenced in 2018.
- Data are extracted weekly from general practices into the Patron database which currently includes over 3.5 million patients from 129 primary care practices across the states of Victoria and Queensland.
- A wide range of de-identified data, including reason for visit, clinical observations (e.g. height weight, blood pressure), diagnosis, investigations, pathology results, medications and referrals are curated for accuracy and consistency by a health informatics team specializing in general practice data, and can be linked to clinical and administrative datasets.
- Data can be used by government, educational institutions, researchers and industry whose applications are approved by an independent data governance group [http://www.gp.unimelb.edu.au/datafordecisions].

## Data resource basics

The Primary Care Audit, Teaching and Research Open Network (Patron) is a growing database of de-identified electronic medical records (EMR) of over 3.5 million patients from 129 consenting Australian general practices. It is a high-quality data repository that provides information at the patient level, and can be linked to administrative and other datasets to provide a better understanding of important health issues. Data from Patron can be used for a broad range of research including analysing the patient journey across primary and secondary care, identifying the type of care received in general practice, monitoring the safety and effectiveness over time of medication and other interventions, measuring randomized controlled trial outcomes, detailing the costs of treatment, and describing health outcomes. It can also be used to understand the patients and conditions that students are exposed to during their primary care clinical placements which, in turn, can facilitate planning and development of the medical curriculum and student placements.

## Scope: Australian general practice

In the Australian health care system, general practitioners (GPs) act as gatekeepers to specialist care, and nearly three-quarters of all medical consultations occur in the primary care setting.^1^ Patients are not obligated to register with an individual doctor or practice and can attend any GP of their choice anywhere in Australia. A national government insurance scheme, called Medicare, covers some or all of the costs for primary care and specialist consultations, investigations and procedures.^2^ Individuals are also encouraged to take out private health insurance for private hospital and ancillary health costs. The majority of Australian general practices use medical records software to record patient data.^3^

## Patron

The technical foundations of Patron were established in the early 2000s when the School of Rural Health and the Department of General Practice at the University of Melbourne (UoM) established an electronic practice-based research network called CONDUIT^4^ and developed the GRHANITE software tool which can extract and link de-identified medical information.^5^ GRHANITE was developed by one of the authors of this paper (DB) and the Health and Biomedical Informatics Centre—Research Information Technology Unit (HaBIC R^2^) at the University of Melbourne. Patron was developed by the Department of General Practice and Primary Care as a strategic venture in collaboration with general practices from its practice-based research network, the Victorian Research and Education Network (VicREN).^6^

The aim of Patron is to increase the use and usability of routinely collected general practice data in research and policy development, and to inform medical training. It is the only Australian primary care database embedded in an academic Department of General Practice and Primary Care and whose development was led by general practitioners (GPs). The Patron database is curated by HaBIC R^2^ who are responsible for managing, harmonizing and sharing the data in accordance with the established Standard Operating Procedures, Ethics, and Data Governance Committee guidelines.

The first wave of 56 general practices were recruited to partner with the University of Melbourne in establishing Patron in early 2018, and data were first shared with researchers later that year. As of December 2022, 129 practices contribute data to Patron, 126 of which are in the state of Victoria and three of which are in the state of Queensland) (Table 1). Of the 129 practices, 103 store their EMRs on a unique server and the remaining 26 practices store their EMRs on one of 10 shared servers, making a total of 113 servers. A server may be shared by practices that are under the same company ownership. Where practices share a server, data are combined and extracted as one ‘site’. Recruitment of practices outside Victoria is currently under way through the PARTNER Network initiative which is a national rural practice-based research network that has been established to support primary care trials.^7^ Practices are not paid for participating in Patron.

**Table 1.** Key features of the Patron database

Feature	Description
Geographical coverage	Patron comprises data from 126 practices in Victoria and 3 practices in Queensland. According to the Modified Monash Model, which classifies a location based on remoteness and population size, 79 practices are in a metropolitan city, 7 are in a regional town, 8 are in a large rural town, 11 are in a medium rural town and 24 are in a small rural town
Practice information	A total of 56 general practices were recruited in 2018, and 129 practices were contributing data to Patron by December 2022. These practices had a total of 113 server ‘sites’, 16% include <5000 patients, 28% include between 5000 and 9999 patients, 35% include between 10 000 and 19 999 patients, 17% include between 20 000 and 49 999 patients and 4% include >50 000 patients
Who is included?	Patron comprises over 3.5 million patients, including 1 910 376 who are marked by the practice as active, 1 632 012 who are marked as inactive or archived and 70 976 who are marked as deceased
Standardization	Patron data have been converted to the Observational Medical Outcomes Partnership (OMOP) Common Data Model [https://www.ohdsi.org/data-standardization/]. This is the most widely used common model internationally, and through its adoption, Patron data can contribute to national and international research at vast scale (100 million participants). As a result of the conversion, data are widely standardised to SNOMED-CT^a^ AU and RxNorm^b^
Existing linkages	Patron has been linked to a wide variety of datasets via national data linkage units, including Australian Institute of Health and Welfare, Centre for Victorian Data Linkage, SA-NT DataLink^c^ and BioGrid. Any datasets available via these organizations can be linked to Patron (e.g. the National Death Index, Pharmaceutical Benefits Scheme, Medicare Benefits Schedule, pathology laboratory databases, Justice and Education, Victorian Admitted Episodes Database, Victorian Emergency Minimum Dataset, Victorian Cancer Registry and Victorian Integrated Non-Admitted Dataset)

a Systematized Nomenclature of Medicine Clinical Terms (SNOMED-CT) is a health terminology which is used as national standard in many electronic medical records. It facilitates consistent sharing of health information across health care settings and the organizing, querying and analysis of health data.

b RxNorm is produced by the National Library of Medicine in the USA and provides normalized names for generic and branded drugs.

c SA-NT Datalink enables data linkage of administrative and clinical datasets in the Northern Territory and South Australia.

Routinely collected data are de-identified and then extracted for all patients in the EMR, including those who are marked by staff at the practice of origin as active, inactive, archived or deceased. Extraction includes any data that have been entered into the EMR since the practice began using the software up to the current day of extraction, thus enabling longitudinal analysis of the patient journey over time. Currently, over 3.5 million patients are represented in Patron, with nearly 2 million marked as ‘active’ patients in the EMR (Table 2). Clinics have posters/leaflets/informing patients that they are participating in Patron and individuals can withdraw their consent to having their data extracted by advising the practice administrative staff.

**Table 2.** Patient characteristics

	All patients		Active		Inactive/archived		Deceased	
	*n*	%	*n*	%	*n*	%	*n*	%
Number of patients	3 613 364	100	1 910 376	52.9	1 632 012	45.1	70 976	2.0
Gender								
Female	1 874 335	51.9	988 709	51.8	850 851	52.1	34 777	49.0
Male	1 646 149	45.6	854 720	44.7	755 984	46.3	35 445	49.9
Other	987	<0.0	838	<0.0	144	<0.0	5	<0.0
Not recorded	91 541	2.5	65 962	3.5	24 830	1.5	749	1.1
Unknown	352	<0.0	147	<0.0	203	<0.0	2	<0.0
Age, years								
<18	471 230	13.0	304 803	16.0	166 215	10.2	212	0.3
18–64	2 483 808	68.7	1 321 591	69.2	1 156 117	70.8	6 100	8.6
>65	586 439	16.2	254 121	13.3	285 819	17.5	46 499	65.5
Not recorded	71 887	2.0	29 861	1.6	23 861	1.5	18 165	25.6
Index of Relative Socio-economic Disadvantage^a^								
Not recorded^b^	111 255	3.1	84 531	4.4	25 616	1.6	10 254	14.4
1 (most disadvantaged)	362 513	10.0	192 992	10.1	160 076	9.8	9146	12.9
2	194 671	5.4	89 967	4.7	95 558	5.9	10 369	14.6
3	325 999	9.0	148 147	7.8	167 483	10.3	8920	12.6
4	362 431	10.0	161 560	8.5	191 951	11.8	5770	8.1
5	280 492	7.8	152 997	8.0	121 725	7.5	4467	6.3
6	380 706	10.5	225 281	11.8	150 958	9.2	6389	9.0
7	398 822	11.0	223 923	11.7	168 510	10.3	4280	6.0
8	279 504	7.7	148 122	7.8	127 102	7.8	4703	6.6
9	500 926	13.9	270 130	14.1	226 093	13.9	6379	9.0
10 (least disadvantaged)	416 045	11.5	212 726	11.1	196 940	12.1	1108	1.6

a The Index of Relative Socio-economic Disadvantage is an index that summarizes economic and social information from the Australian census. This information includes household income, unemployment, disability, overcrowding, lack of car ownership, and ability to speak English well [abs.gov.au].

b Not recorded status includes electronic medical records that did not contain a postcode (which is required to determine Socio-Economic Indexes for Areas decile) or a postcode did not have a Socio-Economic Indexes for Areas decile attributed to it in the Australian Bureau of Statistics data tables.

## Data collected

Data in Patron are extracted from the EMR of consenting practices that use one of the three clinical software packages used by most general practices in Australia, delivered by Best Practice, Medical Director or Zedmed. Data are de-identified and then extracted using GRHANITE software. GRHANITE uses cryptographic hashing to create a ‘signature’ for each patient which cannot be reversed and does not contain any identifying information, such as patient or practitioner names or date of birth.^8^ When required, data can be linked with other data sources (extracted using GRHANITE, e.g. hospital or registry data) using the encrypted signature key for each patient. In instances where it is necessary to re-identify individuals from the dataset, GRHANITE can facilitate this by using a re-identification key held by the general practice where the data were captured. The re-identification key is unique to the instance of GRHANITE that is installed on that specific general practice computer. Examples of the need to re-identify include if it becomes important to provide certain patients about information arising from the research, or a general practice separately consents to be part of an intervention study where individual patients and/or practice staff could be invited to participate.

**Figure 1.**
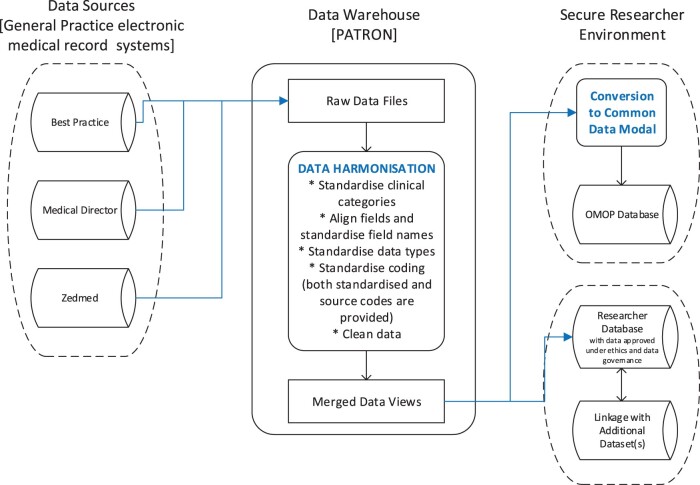
Patron data harmonization to Observational Medical Outcomes Partnership and researcher projects

Following extraction, data from the three different EMR systems are harmonized into a single common format and then organized into a relational database (Figure 1). A description of the data tables and an example of the data fields are shown in Table 3. In addition to general patient information, the data tables include clinical flags, values and dates for ‘phenotypes’ (i.e. a set of data items that identify a cohort of patients) that have been clinically approved. For example, the type 2 diabetes mellitus patient cohort may be described through a clinical diagnosis and/or pathology results. Patron phenotypes have intentionally been constrained to map within a single data category [i.e. Conditions, Pathology, Observations, Medications and Medicare Benefits Schedule (MBS) item numbers] which then become ‘building blocks’ that can be added together to create a clinical algorithm that defines a cohort of patients. These phenotypes can easily be reused to define new cohorts. It is expected that consistent use of these clinical phenotypes across projects will increase the ability of researchers to leverage and build upon existing work.

**Table 3.** Summary of data tables and fields

Table name	Description	Example of fields
Patient details (demographic)		
Patient details	The central table linking with all other tables; it includes the patient’s demographic details	Year of birth, year of death, gender, postcode, indigenous status
Patient details (clinical)		
Observations/measurements	Observations and measurements taken during a visit and recorded by clinic staff	Observation date, type and value (e.g. height, weight, blood pressure)
Allergies	Information on known and observed allergic reactions	Allergen, description of reaction, severity of reaction
Immunizations	Record of immunizations prescribed and/or given to the patient	Immunization date, vaccine, batch number
Smoking	Documents current and historical smoking status, and patient’s desire to quit	Smoking assessment date, status, year started, frequency, quantity consumed, year of cessation (if applicable)
Alcohol consumption	Data on patient’s alcohol consumption using the Alcohol Use Disorders Identification Test (AUDIT-C)	AUDIT-C assessment date, score, consumption details
Cervical screening	Details of cervical screening tests and results	Latest screening date, result of latest screen
Family history		
Family history	Information on status of patient’s parents (i.e. living or deceased) and any significant family history, including relatives	Parents’ age of death and details if applicable, significant clinical conditions of relatives
Encounters		
Encounters	Information about individual visits made by the patient to a general practice	Date of visit, consultation length, age at time of consultation, reason for visit
Medical history		
Medical history	Records previous diagnoses and problems	Condition, diagnosis, age at date of diagnosis, status of condition (active/inactive)
Medications		
Medications	A list of current and previous medications taken by the patient	First and last prescribed dates, name of medication, medication dosage details
Investigations (pathology and imaging)		
Investigations	Details of pathology and imaging results that have been added or imported into the patient’s electronic medical record	Date test performed, date test results reported, test header name, test report
Investigation individual test result details	A record of pathology results (anatomical) extracted from pathology reports that are structured in HL7^a^ format	Test date, name, result, units, results range
Investigation requests	Details investigations ordered by a clinician	Date of request, type of request, name of investigation requested
Document		
Documents	Provides details about documentation received by the clinic (e.g. referrals, discharge summaries, letters) and documentation sent by the clinic (referrals, care plans, medical certificates)	Document date, type, category, subject
Document MyHR	Provides details about shared health summaries and event summaries	Document date, type, category, subject
Services		
MBS billing	A description and information on billings	Service date, MBS item number and description
Practice workforce		
Worker type	Information on the workforce in the practice	Staff roles, prescriber number/provider number/registration number
Maternal obstetrics		
Pregnancies	Details about patient’s current and past pregnancies	Current pregnancy: estimated date of confinement; past pregnancies: delivery date, gestation length, outcome of pregnancy
Antenatal visits	Provides pregnancy observation details that are recorded at each antenatal visit	Baby size in weeks, fetal position

MBS, Medical Benefits Schedule.

a Health Level Seven (HL7) is a set of international standards for the transfer of data between software applications used by various health care providers. In this case it relates to pathology/imaging data being sent from the provider to the general practice.

## Linkage to other datasets

As described above, GRHANITE incorporates a non-reversible cryptographic mechanism to generate de-identified keys for record linkage, commonly known as ‘Hashes’. GRHANITE can extract data from any person-identifiable dataset, allowing probabilistic linkage between them without person identifiers being involved. Patron data have been linked to both national and state-based data repositories, including Australian Institute of Health and Welfare (AIHW), the Centre for Victorian Data Linkage (CVDL), SA-NT Datalink and BioGrid Australia. An example of this is as part of the Victorian Comprehensive Cancer Centre Alliance Data Connect program: Patron data have recently been linked within the CVDL to multiple state-wide datasets including data on hospital admissions, emergency department and out-patient clinic attendances and the Victorian Cancer Registry.

## Data governance, security, privacy and quality

**Figure 2.**
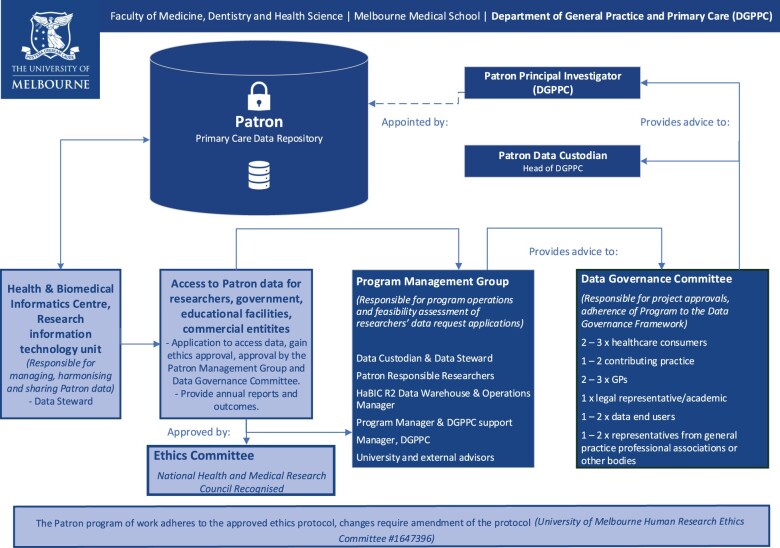
Patron data governance

Patron operates under a rigorous research and data governance framework which includes a program management group and an independent data governance committee, both of which are overseen by the Patron Principal Investigators, Patron Data Steward and the Patron Data Custodian (Figure 2). The database is managed by HaBIC R^2^ and only authorized HaBIC R^2^ staff can access raw data.

The suite of virtual machines and databases that comprise Patron are known as the ‘Patron Data Enclave’. The Patron Data Enclave has been established to provide tight restrictions around access to the data, including the use of multi-factor authentication. The enclave acts as a ‘secure research environment’ limiting the ability for data to move in and out of the enclave, including restricting internet access and prohibiting attached external data storage, except by curated mechanisms. HaBIC R^2^ works with the University of Melbourne Cybersecurity team, and the enclave last completed a successful round of independent penetration testing (i.e. ethical hacking) in July 2022.

Although GHRANITE does not extract fields known to normally contain patient names and birth dates, in reality GPs or practice staff sometimes insert person-identifiable information in unexpected places within the clinical record. To ensure that this information is not included in the data cut provided to researchers, an automated algorithm reviews data in textual fields of the database to screen for the inadvertent presence of names or other person-identifying information. In addition, HaBIC R^2^ undertakes regular manual scrutiny for any mistaken importation of identifiable information.

## Data resource use

To date, 36 projects have used Patron data, eight of which have used linkages to external datasets. Completed projects include clinical trials, epidemiological studies, data quality investigations, and evaluations of interventions implemented in an individual, or group of, participating practices. For example, Patron data have been used to better understand antibiotic prescribing in general practice,^9^ the impact of the COVID-19 pandemic on general practice activity^10^ and to explore whether electronic medical record data can be used to monitor medicinal cannabis prescribing.^11^ Detailed information on projects that have been approved to use Patron data, and the peer-reviewed publications arising from these studies, can be found at [https://medicine.unimelb.edu.au/school-structure/general-practice/engagement/data-for-decisions#projects].

## Strengths and weaknesses

### Strengths

A key strength of Patron is its ability to accurately capture the general practice activity of participating practices and provide useable data to approved researchers. Unlike other primary care datasets in Australia, data in Patron are curated by a specialist general practice research information technology unit (i.e. HaBIC R^2^) who clean, code and standardize the complex, and often un-coded, raw data entered by clinicians. Confidence in the quality of the data, combined with a longitudinal dataset comprising over 3 million patients, means that Patron data can be used in clinical trials, epidemiological studies and surveillance studies and make a real-world difference in care processes and patient outcomes. Another strength is that because data are de-identified and have proactive measures around ongoing de-identification, Patron meets the requirements for research to be undertaken with a waiver of patient consent, which increases the feasibility of population-based studies. Additionally, rather than researchers having to contact a practice for consent for each new data-led study, the independent Patron Data Governance Committee, comprising consumers, GPs, practice managers, a lawyer, information technology (IT) specialist, can approve data requests on behalf of the contributing practices for projects that have appropriate research ethics committee approvals. This is a time and resource saving for research teams, which ultimately facilitates high-quality outputs.

### Weaknesses

Although Patron includes over 3.5 million patients, the practices that contribute data are concentrated in Victoria, which is the most urbanized state in Australia. Therefore, the data may not be representative of general practices across the country and there are limitations in the assumptions that can be made about the population as a whole. In particular, Patron is not representative of First Nations Australians. This is because Victoria has a lower proportion of First Nations Australians than some other states and because recording of First Nations status in the EMR is sub-optimal.^12^ More broadly, ethnicity is sub-optimally recorded. In order to ensure that this is understood and considered by data users, our informatics team carry out feasibility assessments for data requests and, together with feedback from the Patron management group, provide feedback to the data user. Another limitation is the difficulty in capturing a patient’s activity if they also attended a practice that is not a Patron partner.

Issues with data extraction have also occurred when there have been IT infrastructure changes in the practice, or the computer used to extract the data is turned off. However, this can be rectified once the issue has been identified and new data entered by the practice during the pause can be retrospectively extracted.

### Data resource access

Patron data can be used by researchers, government, educational institutions and commercial entities following approval from Patron’s independent Data Governance Committee and a National Health and Medical Research Council approved ethics committee. The Data Governance Committee is responsible for ensuring that proposed projects are within the scope agreed to by participating practices and that projects do not duplicate each other.^13^ Researchers are required to provide a data risk and management plan as part of their application.

When practices sign up to Patron, they are asked to indicate consent as to whether they agree for data from their practice to be used in research funded by and/or conducted by commercial entities. Most practices (87.6%) have consented to commercially funded research and about half (50.4%) to research undertaken by a commercial entity. Commercial entities must agree that researchers retain the right to publish research findings regardless of the study outcome.

To ensure that the interpretation of Patron data accurately reflects general practice activity, a GP or primary care academic must be included in the data users’ research team. The role of the GP or primary care academic is to provide contextual knowledge about the likely quality of data entry in each field of the EMR extracted, to advise on the limitations of the data and to ensure that journal articles based on the data include appropriate descriptions of Patron and draw reasonable conclusions.

For each approved project, a data cut based on the project data requirements is provided to the researcher. The data cut is informed by the principle of ‘data minimization’, with researchers only provided with the fields necessary to complete the study described in their approved application.

## Ethics approval

Patron has ethics approval from the University of Melbourne Human Research Ethics Committee (HREC 1647396; 23358) to recruit general practices and extract non-identifiable patient data from their EMRs using the GRHANITE data extraction tool. Individual patient consent is not required; however, practices must display information about their participation in Patron in a location that is easily viewed by patients. Patients who do not want their data extracted can advise the practice who must then record the patient as ‘withdrawn’, either directly into GRHANITE or into the EMR if it provides this functionality. No further data will be extracted for that patient and previously extracted data will be withdrawn from the database. Data that have already been provided to researchers cannot be withdrawn.

## Data Availability

See ‘Data Resource Access’, above.
